# Association Between Radiological Stenosis Level and Patient-Reported Outcomes in Patients with Lumbar Spinal Stenosis: A Cross-Sectional Study

**DOI:** 10.3390/medicina62010029

**Published:** 2025-12-23

**Authors:** Selda Çiftci İnceoğlu, Aylin Ayyıldız, Bora Şahin, Sefa Özcan, Alperen İnceoğlu, Hakan Ayyıldız, Banu Kuran

**Affiliations:** 1 Department of Physical Medicine and Rehabilitation, Health Sciences University, Şişli Hamidiye Etfal Training and Research Hospital, İstanbul 34371, Turkey; 2 Department of Physical Medicine and Rehabilitation, Başakşehir Çam & Sakura City Hospital, İstanbul 34480, Turkeyalperenatvs@gmail.com (A.İ.); 3 Department of Radiology, Başakşehir Çam & Sakura City Hospital, İstanbul 34480, Turkey; hakanayyildiz77@gmail.com

**Keywords:** cross-sectional study, lumbar spinal stenosis, patient-reported outcomes, radiology

## Abstract

*Background and Objectives*: The aim of this study was to evaluate the relationship between low back pain questionnaires and radiological stenosis severity in patients with lumbar spinal stenosis (LSS). *Materials and Methods*: Patients aged 50 years and over who presented with complaints of low back pain and were diagnosed or not diagnosed with LSS by magnetic resonance imaging (MRI) were included in the study. Demographic data, physical examination findings, and walking distance were recorded. Pain severity was assessed using the Visual Analog Scale (VAS), and patients completed the Oswestry Disability Index (ODI), the Istanbul Low Back Pain Disability Index (ILBPDI), and the Swiss Spinal Stenosis Questionnaire (SSS-Q). *Results*: A total of 120 patients with LSS (*n* = 56) and without LSS (*n* = 64) were included in the study. No significant differences were found between the groups in terms of demographic variables (*p* > 0.05). Neurogenic claudication and lumbar extension limitation were higher in the LSS group (*p* = 0.033 and *p* = 0.008, respectively), and walking distance was significantly shorter compared to the group without LSS (*p* = 0.024). There were significant differences between the VAS, ODI, ILBPDI, and SSS-Q scores between the two groups (*p* < 0.05). A strong positive correlation exists between the radiological severity of LSS and SSS-Q (*p* < 0.001, r = 0.707). Additionally, ROC analysis revealed that the SSS-Q had a significantly higher diagnostic value for LSS compared to the ODI and ILBPDI (*p* < 0.001). For the SSS-Q, likelihood ratios indicated limited diagnostic relevance (PLR 4.04 [95% CI: 2.45–6.67]; NLR 0.22 [95% CI: 0.13–0.44]). *Conclusions*: SSS-Q, ODI, and ILBPDI scores vary significantly between patients with and without LSS. Although the SSS-Q correlates most strongly with radiological LSS severity, its diagnostic utility appeared of minor importance, as likelihood ratios indicated limited discriminative ability.

## 1. Introduction

Lumbar spinal stenosis (LSS) is a major cause of disability in older adults, characterized by narrowing of the spinal canal in the lumbar region. Despite the fact that LSS has a high global prevalence, there is currently no universally accepted definition or standardized radiological diagnostic criteria for the condition [1]. LSS can affect the central canal, lateral recess, neural foramen, or extraforaminal region [2]. Central stenosis, most commonly at the L4–L5 level, is usually caused by ligamentum flavum hypertrophy and posterior disc bulging. Lateral recess stenosis is a condition that arises from facet joint arthropathy and osteophyte formation, resulting in nerve compression prior to the foramen. Foraminal stenosis arises from disc height loss, disc protrusion, or osteophytes, affecting the nerve within the foramen. Extraforaminal stenosis, which is typically caused by far lateral disc herniation, results in the compression of the nerve root after its exit from the foramen [3].

LSS can be categorized as either congenital or acquired. The most prevalent form is degenerative spondylosis, which develops due to factors such as trauma, aging, and long-term mechanical loading and stress [1]. The lack of universally accepted diagnostic criteria for LSS makes epidemiological assessment difficult. However, one systematic review reported a prevalence of 11% in the general population and 25–39% in the clinical setting [4]. Magnetic resonance imaging (MRI) is the preferred method for LSS. There are various MRI-based classification methods [1]. The Lee grading system is a classification system based on the cerebrospinal fluid (CSF) distance and the shape of the cauda equina [5]. This classification system provides practical tools for assessing patients’ stenosis and developing appropriate treatment plans [6]. Initial management in LSS treatment usually involves conservative treatment options, and surgical intervention may be considered when these approaches fail. Minimally invasive surgical approaches are preferable because they are associated with shorter hospital stays and lower risks compared to fusion or instrumentation surgery [7].

As with many musculoskeletal diseases, the use of patient-reported outcomes (PROs) is widespread in low back pain and serves to assess symptom severity, daily functioning, and treatment outcomes. These questionnaires may include the Roland Morris Disability Questionnaire, the Oswestry Disability Index (ODI), the Quebec Back Pain Disability Scale, and the Istanbul Low Back Pain Disability Index (ILBPDI) [8,9]. General pain questionnaires are also used to assess low back pain [8]. The Swiss Spinal Stenosis Questionnaire (SSS-Q) is a disease-specific PROs that includes questions about the symptoms of spinal stenosis [10].

The aim of this study was to evaluate the correlation and diagnostic performance of PROs developed for low back pain in patients with LSS in relation to the degree of radiological stenosis. In addition, the association between physical examination findings and radiological stenosis severity was examined.

## 2. Materials and Methods

### 2.1. Study Design

This study was conducted as a cross-sectional design, with all assessments completed during a single visit. The study was completed in the outpatient clinic of Physical Medicine and Rehabilitation (PM&R) of Şişli Hamidiye Etfal Training and Research Hospital between 1 June and 1 September 2025. Ethics committee approval for the study was received from the Ethics Committee of Şişli Hamidiye Etfal Training and Research Hospital (8 April 2025/approval no: 4828). The study is registered at clinicaltrails.gov (NCT07058350). Informed consent was obtained from all participants, and this study complied with the principles outlined in the Declaration of Helsinki.

### 2.2. Participants

Patients who applied to the PM&R outpatient clinic with complaints of low back pain, were aged 50 years or older, and had lumbar MRI within the last year were included in the study. Patients were evaluated for LSS using history, physical examination, and MRI. Exclusion criteria included congenital, traumatic, or iatrogenic lumbar spinal stenosis, a history of lumbar surgery, severe hip, knee, or ankle disease, and cognitive impairment. Lumbar spine MRI was performed using a 1.5 Tesla system with axial T2-weighted fast spin-echo sequences. An experienced, blinded radiologist evaluated the presence and severity of LSS at the L4-5 level, the most common site of degenerative LSS, using the Lee grading system [5]. According to this classification, stenosis is graded based on anteroposterior narrowing observed on MRI: Grade 0 indicates no stenosis, Grade 1 mild stenosis, Grade 2 moderate stenosis, and Grade 3 severe stenosis. Participants were divided into two groups: those with and those without LSS. In this study, only patients with Grade 0 according to the Lee grading system were included in the non-LSS group, while those with Grade 1-2-3 were included in the LSS group. Additional levels and compartments were also systematically evaluated to determine the full extent of LSS. These assessments were used for descriptive analyses and to determine whether stenosis was present at a single level or multiple levels. Single stenosis was classified as the stenosis being limited to only one lumbar level and one compartment, and multiple stenosis was classified as the stenosis being at ≥2 lumbar levels and/or affecting more than one compartment (central canal, lateral recess, foraminal). Fatty degeneration of the paravertebral muscles was graded into five stages based on the degree of fatty infiltration. Grade 0 indicates normal muscle with no fatty deposits. Grade I indicates the presence of minimal focal or linear fat within the muscle. Grade II involves up to 25% fatty infiltration, while Grade III corresponds to 25% to 50% fat content. Grade IV indicates greater than 50% fatty infiltration [11].

### 2.3. Clinical Assessment

Patient demographics, duration of low back pain, presence of additional symptoms and walking distance were assessed. Physical examination was performed with lumbar joint range of motion and manual muscle strength testing. Walking distance was determined by asking patients to report the distance they were able to walk before back or leg symptoms occurred and symptoms forced them to stop. The Timed Up and Go (TUG) test was administered by having participants stand up from the chair, walk 3 m, return, and sit back down in a chair, all recorded using a chronometer. The TUG test also provides objective functional assessment in patients with LSS [12]. Patients were questioned about their pain levels using a Visual Analog Scale (VAS). They were asked to mark their pain level on a 10 cm ruler. 0 mm indicates no pain, and 100 mm indicates the most severe pain [13].

One of the questionnaires in the study, ODI, is used to measure the degree of functional limitations caused by pain in patients with low back pain. The ODI provides detailed information about a patient’s overall daily functioning. Questions include pain intensity, self-care, lifting, moving, sitting or standing, sleep, sex, social life, and travel/transportation. The patient is asked to rate the level of disability on a scale of 0 to 5. The index is calculated by dividing the total score by the range of scores and multiplying the result by 100. A score of 0% indicates no disability, while 100% indicates the highest level of disability [14,15].

The ILBPDI consists of 18 questions that assess the effects of low back pain on activities such as climbing stairs, walking, bathing, brushing teeth, dressing, etc. Individuals are asked to score these activities on a scale of 0 to 5, based on their level of difficulty. A total score of 0 indicates no disability, while a score of 90 indicates maximum disability [9].

The SSSQ is a condition-specific instrument comprising 18 items across three domains: symptom severity (7 items), physical function (5 items), and treatment satisfaction (6 items). Symptom severity includes assessments of pain (intensity, frequency, and back pain) and neuroischemic complaints (numbness/tingling, weakness, balance problems), each scored on a 1–5 scale (max 35). Physical function and satisfaction domains are scored on a 1–4 scale, with higher scores indicating greater disability and lower satisfaction, respectively [10].

### 2.4. Bias

To minimize potential bias associated with the cross-sectional design, all assessments were performed by a single physician blinded to the participants’ diagnoses and MRI results.

### 2.5. Sample Size

Sample size was calculated using G*Power (version 3.1.9.7). Based on a medium effect size (d = 0.5), 80% power, and a 5% significance level, a total of 128 participants (64 per group) were required.

### 2.6. Statistical Analysis

Statistical analyses were conducted using jamovi software (Version 2.5; The jamovi project, 2024) [Computer Software], available at ‘https://www.jamovi.org (accessed on 19 September 2025)’. Descriptive statistics were reported as mean ± standard deviation, median (interquartile range), frequencies, and percentages, as appropriate. The distribution of continuous variables was assessed using histograms and the test. For comparison of independent quantitative variables, the Student’s *t*-test or Mann–Whitney U test was used based on distribution. Categorical variables were analyzed using the chi-square test or Fisher’s exact test, as appropriate. Correlation between variables was evaluated using Spearman’s rank correlation coefficient. ANOVA and Kruskal–Wallis tests were used for comparisons of more than two groups. Tukey test and Dwass–Steel–Critchlow–Fligner (DSCF) pairwise comparison analyses were performed for significant values. Bonferroni correction was applied for within-group analyses.

## 3. Results

**Figure 1:**
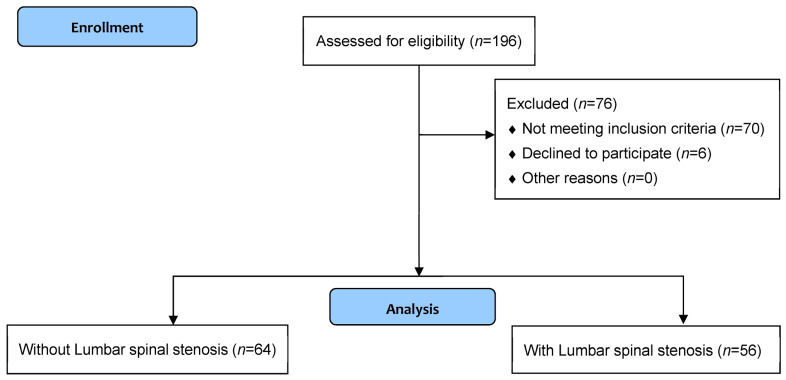
Flow diagram of study (Adapted from CONSORT 2010 Flow Diagram).

One hundred and ninety-six patients presenting to the PM&R clinic with complaints of low back pain were evaluated according to the inclusion criteria. The study was completed with a total of 120 patients: 56 with LSS and 64 without LSS (Figure 1). No significant differences were found between the groups in terms of age, gender, education level, presence of comorbidities, smoking status, occupational intensity, duration of low back pain, or presence of fatty degeneration on MRI (*p* > 0.05). However, limited lumbar extension and neurogenic claudication were significantly higher in the LSS group (*p* = 0.008 and *p* = 0.033, respectively). Walking distance was significantly shorter in the LSS group than in the non-LSS group, and the TUG test was significantly prolonged (*p* = 0.024 and *p* = 0.023, respectively) (Table 1 and Table 2).

Significant differences were found between the LSS and non-LSS groups in terms of VAS, ODI, SSS-Q total score, SSS-Q symptom score, SSS-Q physical score, and ILBPDI scores; scores were significantly higher in the LSS group in all of these assessments (*p* < 0.05). Furthermore, no significant differences were found when comparing patients with LSS in terms of VAS, ODI, SSS-Q total score, SSS-Q symptom score, SSS-Q physical score, and ILBPDI scores based on the presence of single or multiple stenoses (*p* > 0.05) (Table 2).

One of the objectives of this study was to determine whether differences existed between the degree of LSS and pain severity as well as PROs measure. There was a significant difference between the groups in terms of VAS scores (*p* < 0.001). According to the radiological Lee grading system confirmed by MRI, the VAS scores of the mild LSS group were significantly different from both the moderate and severe LSS groups (*p* = 0.018, *p* < 0.001, respectively). However, there was no significant difference in VAS between the moderate and severe LSS groups (*p* = 0.103). ODI scores also differed significantly according to stenosis level in patients with LSS (*p* = 0.001). In pairwise comparisons performed to determine the origin of this difference, a significant difference was found only between mild and severe LSS (*p* = 0.002, DCSF pairwise comparison). There were significant differences between the SSS-Q total scores, symptom severity scores, and physical scores of the LSS groups according to severity level (*p* < 0.001). There were also significant differences in ILBPDI scores among the groups determined according to stenosis level (*p* < 0.001). There was no significant difference in ILBPDI scores between the mild and moderate LSS groups (*p* = 0.495). However, there was a significant difference between the mild and severe LSS groups and the moderate and severe LSS groups (*p* < 0.001, *p* = 0.039, respectively). Comparisons of pain and PROs according to lumbar spinal stenosis severity are shown in Table 3.

PROs is used for many purposes, such as determining the severity of patients’ symptoms and monitoring treatment responses. The relationship between these questionnaires was also evaluated in this study. A very high positive correlation was found between ODI and ILBPDI scores (*p* < 0.001, r = 0.897). There was a high positive correlation between ODI and SSS-Q scores and between ILBPDI and SSS-Q scores (*p* < 0.001, r = 0.657; *p* < 0.001, r = 0.724, respectively). Another important result was the presence of a high positive correlation between SSS-Q and the Lee grading system (*p* < 0.001, r = 0.707). A weak positive correlation was found between the ODI and Lee grading system (*p* < 0.001, r = 0.390), and a moderate positive correlation was found between the ILBPDI and Lee grading system (*p* < 0.001, r = 0.415).

**Figure 2:**
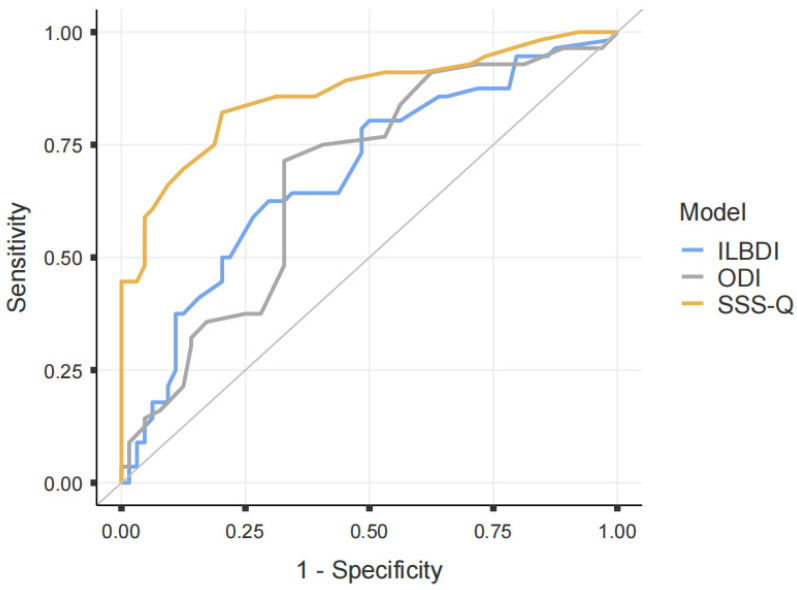
ROC analysis of patient-reported outcomes. Sensitivity; True positive rate, 1-specifity; false positive rate.

The diagnostic value between the LSS and PROs was also examined in this study. Receiver operating characteristic (ROC) analysis revealed area under curve (AUC) values of 0.861 for the SSS-Q, 0.678 for the ODI, and 0.686 for the ILBPDI. Comparisons were made using the Delonge test. The diagnostic value of the SSS-Q was significantly higher than that of the ODI and ILBPDI (*p* < 0.001), and there was no significant difference between the ODI and ILBPDI (*p* = 0.754) (Figure 2). Considering the AUC values, which were above 0.80, its diagnostic value can be considered high. However, it is important not to draw conclusions based on the AUC value alone. Therefore, it is necessary to examine the positive likelihood ratio (PLR) or negative likelihood ratio (NLR) values [16]. For the SSS-Q, the PLR was 4.04 (95% CI: 2.45 to 6.67) and the NLR was 0.22 (95% CI: 0.13 to 0.44). Accordingly, it can be of little or no clinical diagnostic significance.

## 4. Discussion

This study investigated the relationship between clinical and radiological LSS and patient-reported measures. Significant differences were observed in SSS-Q, ODI, and ILBPDI scores between patients with and without LSS. Among the evaluated questionnaires, the SSS-Q showed greater diagnostic relevance than the ODI and ILBPDI; however, this finding was not supported by PLR and NLR analyses. This indicates that, although the SSS-Q outperforms other PROs in relative terms, its overall diagnostic utility remains limited, and it should not be considered a stand-alone diagnostic tool for LSS.

Degenerative spinal diseases, such as disc space narrowing, degenerative spondylolisthesis, adult spinal deformities, or lumbar spinal stenosis, can lead to pain and disability [7]. LSS is generally associated with lower back pain that is aggravated by walking and lumbar extension, radiculopathy, and neurogenic claudication [1]. This may explain the observed difference in lumbar extension limitation between the groups in the study, as lumbar extension in patients with lumbar spinal stenosis can be restricted not only by pain during examination but also by degenerative factors. Neurogenic claudication in spinal stenosis primarily results from central spinal canal narrowing, leading to multi-level compression of the cauda equina and venous occlusion, resulting in decreased arterial perfusion during standing and walking, and ischemia of the nerve roots [17]. Therefore, in this study, where patient groups were formed according to the presence of central stenosis, the higher incidence of neurogenic claudication in the LSS group is an expected result. The relationship between LSS and walking ability has been investigated in studies [18,19]. As expected, in this study, patients with LSS had a shorter walking distance and a longer duration of the TUG test.

PROs is valuable for clarifying symptom severity and monitoring treatment outcomes across various conditions. It is also used in long-term follow-ups at LSS [20]. In low back pain, both low back pain-specific and general PROs is frequently used. A recently published consensus statement includes nine recommended clinical outcome measures for LSS. Walking distance, VAS, ODI, and SSS-Q are among those recommended in the consensus and used in this study [21]. In the literature, the relationship between the ODI and the severity of symptoms or the degree of stenosis on MRI has been frequently compared. However, in these studies, either no correlation or a weak correlation was generally found [22,23,24,25,26,27,28,29]. This may be due to the fact that clinical symptoms do not always increase in parallel with the degree of stenosis or that ODI is not a test for the cause, type and diagnosis of pain. In a cohort study by Yeung et al. [30], ODI scores did not differ significantly between patients with no or mild LSS and those with moderate to severe stenosis, although both groups showed significant postoperative improvement. This suggests that PROs may be more useful for monitoring treatment outcomes than for grading radiologic stenosis severity.

The ILBPDI is a low back pain questionnaire developed in our country. It has been used in studies to evaluate the outcomes of LSS before and after epidural steroid treatment [31,32]. It has been stated that the ILBPDI, ODI, Back Pain Functional Scale, and Quebec Back Pain Disability Scale can be used to assess disability in cases of chronic mechanical low back pain, and may even be used interchangeably due to the strong correlations among them [33]. Consistent with this, this study demonstrated a very high correlation between the ODI and ILBPDI. As with the ODI, the ILBPDI cannot provide clear information about the type and cause of low back pain; it may be more suitable for the evaluation of disability due to low back pain.

The SSS-Q is a PROs developed specifically for LSS [10]. This study demonstrates a strong correlation between the radiological stenosis level and SSS-Q scores. While all three questionnaires (ODI, ILBPDI, and SSS-Q) demonstrated significant differences between the LSS and non-LSS groups, the stronger correlation between the SSS-Q and the radiological stenosis severity in patients with stenosis reveals that the SSS-Q may be more suitable for assessing patients with LSS. In a study evaluating LSS patients before and after surgery, SSS-Q, ODI, and Oxford Limping Score (OCS) performances were similar, but SSS-Q was found to be the most sensitive [34]. In a study conducted as a secondary analysis of a randomized controlled trial, it was shown to be sensitive for use in patients with non-operated LSS [35]. To our knowledge, no studies have evaluated the ODI, ILBPDI, and SSS-Q together in patients with LSS, highlighting the potential value of the present study for informing future research.

This study has several limitations, including being a single-center study with a relatively small sample size of patients aged 50 years and older with low back pain and not assessing the effects of common pain syndromes and other causes of low back pain —such as facet joint arthropathy, sacroiliac joint dysfunction, and osteoporotic changes— on PROs. Furthermore, the study’s cross-sectional design may preclude causal inference, and the lack of external validation or longitudinal results may significantly limit the generalizability and direct clinical impact of the ROC-based findings. Other limitations of the study are that MRI examiner blinding reduces but may not eliminate measurement bias, and self-report questionnaires may still be subject to recall and reporting bias. Additionally, although the final sample included 120 participants instead of the planned 128, significant differences in PROs were still observed between patients with and without LSS, confirming that the study was adequately powered to detect meaningful effects. PLR and NLR were reported instead of positive and negative predictive values in the statistical analysis, thereby avoiding potential inaccuracies arising from prevalence. Future studies involving multiple centers, larger patient populations, and longer-term follow-up are needed to support the findings of this study.

## 5. Conclusions

SSS-Q, ODI, and ILBPDI scores differ significantly between patients with and without lumbar spinal stenosis. While the SSS-Q exhibits the strongest correlation with radiological stenosis severity, likelihood ratio analyses indicate that its overall diagnostic utility is limited.

## Figures and Tables

**Table 1:** Demographic variables and physical examination of patients.

		Non-LSS (*n* = 64)	LSS (*n* = 56)	*p*
Age (year)	Median (IQR)	60.0 (IQR = 12.25)	61.0 (IQR = 18.25)	0.302 ^m^
Gender:				
Female	*n* (%)	50 (41.7%)	44 (36.7%)	0.953 ^x^
Male		14 (11.7%)	12 (10.0%)	
Occupation:				
Sedentary	*n* (%)	56 (46.7%)	47 (39.2%)	0.325 ^x^
Light/moderate		8 (6.7%)	8 (6.7%)	
Heavy		0 (0.0%)	1 (0.8%)	
Additional disease:				
Yes	*n* (%)	35 (29.2%)	29 (24.2%)	0.751 ^x^
Essential hypertension:				
Yes	*n* (%)	27 (22.5%)	25 (20.8%)	0.787 ^x^
Diabetes mellitus:				
Yes	*n* (%)	13 (10.8%)	8 (6.7%)	0.386 ^x^
Coronary artery disease:				
Yes	*n* (%)	10 (8.3%)	20 (8.3%)	0.743 ^x^
Cigarette usage:				
Yes	*n* (%)	17 (14.2%)	11 (9.2%)	0.371 ^x^
Duration of low back pain (months)	Median (IQR)	24 (IQR = 36.0)	18.0 (IQR = 42.0)	0.187 ^m^
Pain radiating to the lower extremities:				
Yes	*n* (%)	39 (32.5%)	43 (35.8%)	0.063 ^x^
Limited lumbar extension:				
Yes	*n* (%)	28 (23.3%)	38 (31.7%)	0.008 ^x^
Neurogenic claudication:				
Yes	*n* (%)	10 (8.3%)	18 (15.0%)	0.033 ^x^
Muscle weakness:				
Yes	*n* (%)	7 (5.8%)	10 (8.3%)	0.278 ^x^
Walking distance (m)	Median (IQR)	500.0 (IQR = 400.0)	450.0 (IQR = 425.0)	0.024 ^x^
Timed up and Go Test (s)	Median (IQR)	10.0 (IQR = 3.0)	11.5 (IQR = 5.25)	0.023 ^x^

^X^ chi-square test, ^m^ Mann–Whitney U test. LSS; lumbar spinal stenosis, IQR; interquartile range.

**Table 2:** Comparison of patients’ magnetic resonance imaging and patient-reported outcomes.

		Non-LSS (*n* = 64)	LSS (*n* = 56)	*p*
Lee grading system				
Grade 0		64 (53.3%)	-	
Grade 1		-	24 (20.0%)	
Grade 2		-	11 (9.2%)	
Grade 3	*n* (%)	-	21 (17.5%)	-
Lee grading system	Median (IQR)	0.0 (IQR = 0.0)	2.0 (IQR = 2.0)	<0.001 ^m^
Fatty degeneration of paravertebral muscle				
0		10 (8.3%)	11 (9.2%)	
1		13 (10.8%)	15 (12.5%)	
2		15 (12.5%)	10 (8.3%)	
3		12 (10.0%)	16 (13.3%)	
4	*n* (%)	1 (0.8%)	4 (3.3%)	-
Fatty degeneration of paravertebral muscle	Median (IQR)	2.0 (IQR = 1.0)	2.0 (IQR = 2.0)	0.803 ^m^
VAS	Median (IQR)	4.0 (IQR = 2.0)	6.0 (IQR = 3.0)	<0.001 ^m^
ODI (score)	Median (IQR)	30.0 (IQR = 20.5)	38.0 (IQR = 18.5)	<0.001 ^m^
ODI (%)				
0–20%		7 (5.8%)	2 (1.7%)	
20–40%		36 (30.0%)	27 (22.5%)	
40–60%		18 (15.0%)	20 (16.7%)	
60–80%		3 (2.5%)	6 (5.0%)	
80–100%	*n* (%)	0 (0.0%)	1 (0.8%)	0.033 ^m^
ILBPDI	Median (IQR)	31.5 (IQR = 13.25)	41.0 (IQR = 15.25)	<0.001 ^m^
SSS-Q total score	Median (IQR)	19.0 (IQR = 7.0)	30.0 (IQR = 14.0)	<0.001 ^m^
SSS-Q Symptom severity score	Median (IQR)	11.0 (IQR = 5.0)	20.0 (IQR = 12.0)	<0.001 ^m^
SSS-Q Symptom severity average				
1–2		53 (44.2%)	13 (10.8%)	
2.1–3		11 (9.2%)	19 (15.8%)	
3.1–5	*n* (%)	0 (0.0%)	24 (20.0%)	<0.001 ^m^
SSS-Q Physical function score	Median (IQR)	7.0 (IQR = 4.0)	10.0 (IQR = 4.25)	<0.001 ^m^
SSS-Q Physical function average				
1–1.5		35 (29.2%)	10 (8.3%)	
1.6–2.5		27 (22.5%)	27 (22.5%)	
2.6–4	*n* (%)	2 (1.7%)	19 (15.8%)	<0.001 ^m^

^m^ Mann–Whitney U test. LSS; lumbar spinal stenosis, VAS; Visual Analog Scale, ODI; Oswestry Disability Index, ILBPDI; Istanbul Low Back Pain Disability Index, SSS-Q; Swiss Spinal Stenosis Questionnaire.

**Table 3:** Comparison of pain and patient-reported outcomes according to lumbar spinal stenosis severity.

		Grade 1 LSS (*n* = 24)	Grade 2 LSS (*n* = 11)	Grade 3 LSS (*n* = 21)	*p*
VAS	Mean ± SD	4.33 ± 1.58	5.91 ± 1.58	7.10 ± 1.45	<0.001 ^a^
ODI	Mean ± SD	34.5 ± 13.35	38.23 ± 8.5	49.43 ± 13.51	*p* = 0.001 ^k^
ILBDI	Mean ± SD	35.96 ± 12.52	41.0 ± 6.42	52.38 ± 13.81	<0.001 ^a^
SSS-Q total score	Mean ± SD	25.08 ± 7.56	30.55 ± 7.22	38.57 ± 6.49	<0.001 ^a^
SSS-Q Symptom severity score	Mean ± SD	16.29 ± 5.19	19.45 ± 4.7	25.61 ± 4.81	<0.001 ^a^
SSS-Q Physical function score	Mean ± SD	8.79 ± 2.96	11.09 ± 1.92	12.95 ± 2.36	<0.001 ^a^

^a^ ANOVA test, ^k^ Kruskal–Wallis test. Bonferroni correction was applied to *p*-values. LSS; lumbar spinal stenosis, VAS; Visual Analog Scale, ODI; Oswestry Disability Index, ILBPDI; Istanbul Low Back Pain Disability Index, SSS-Q; Swiss Spinal Stenosis Questionnaire.

## Data Availability

The data associated with the paper are not publicly available but are available from the corresponding author on reasonable request.

## Abbreviations

AUC: Area under curve, ILBPDI: Istanbul Low Back Pain Disability Index, LLS: lumbar spinal stenosis, MRI: magnetic resonance imaging, ODI: Oswestry Disability Index, PM&R: Physical Medicine and Rehabilitation, ROC: Receiver operating characteristic, SSS-Q: Swiss Spinal Stenosis Questionnaire, VAS: Visual Analog Scale.
